# Phloroglucinol Attenuates Motor Functional Deficits in an Animal Model of Parkinson's Disease by Enhancing Nrf2 Activity

**DOI:** 10.1371/journal.pone.0071178

**Published:** 2013-08-20

**Authors:** Junghwa Ryu, Rui Zhang, Bo-Hyun Hong, Eun-Jung Yang, Kyoung Ah Kang, Moonseok Choi, Ki Cheon Kim, Su-Jin Noh, Hee Soo Kim, Nam-Ho Lee, Jin Won Hyun, Hye-Sun Kim

**Affiliations:** 1 Department of Pharmacology and Biomedical Sciences, College of Medicine, Seoul National University, Seoul, Republic of Korea; 2 School of Medicine and Institute for Nuclear Science and Technology, Jeju National University, Jeju, Republic of Korea; 3 School of Animal Bioscience and Technology, Konkuk University, Seoul, Republic of Korea; 4 Department of Chemistry, College of Natural Sciences, Jeju National University, Jeju, Republic of Korea; 5 Seoul National University Bundang Hospital, Seongnam, Republic of Korea; 6 Seoul National University, Cancer Research Institute, Seoul, Republic of Korea; National Institute of Health, United States of America

## Abstract

In this study, we investigated whether phloroglucinol (1, 3, 5 - trihydroxybenzene) has therapeutic effects in cellular and animal model of Parkinson's disease (PD). PD is the second most common, chronic and progressive neurodegenerative disease, and is clinically characterized with motor dysfunctions such as bradykinesia, rigidity, postural instability, gait impairment, and resting tremor. In the brains of PD patients, dopaminergic neuronal loss is observed in the *Substantia nigra*. Although the exact mechanisms underlying PD are largely unknown, mitochondrial dysfunction and oxidative stress are thought to be critical factors that induce the onset of the disease. Here, phloroglucinol administration was shown to attenuate motor functional deficits evaluated with rota-rod and apomorphine-induced rotation tests in 6-hydroxydopamine (6-OHDA)-induced PD animal models. Moreover, phloroglucinol ameliorated the loss of synapses as assessed with protein levels and immunoreactivity against synaptophysin in the midbrain region of the 6-OHDA-lesioned rats. In addition, in SH-SY5Y cultures, the cytotoxicity of 6-OHDA was reduced by pre-treatment with phloroglucinol. The increase in the reactive oxygen species, lipid peroxidation, protein carbonyl formation and 8-hydroxyguanine caused by treatment with 6-OHDA was attenuated by phloroglucinol in SH-SY5Y cells. Furthermore, phloroglucinol treatment rescued the reduced levels of nuclear Nrf2, antioxidant enzymes, i.e., catalase and glutathione peroxidase, in 6-OHDA-treated cells. Taken together, phloroglucinol has a therapeutic potential for treatment of PD.

## Introduction

Parkinson's disease (PD) is a progressive neurodegenerative disorder mainly characterized by the loss of dopaminergic neurons in the midbrain and the presence, in the affected brain regions, of protein inclusions called Lewy bodies [1]. In the brains of PD patients, selective dopaminergic neuronal loss is observed in the *Substantia nigra* and causes motor dysfunctions such as bradykinesia, rigidity, postural instability, gait impairment, and resting tremor [1]–[3].

In this study, we investigated whether phloroglucinol (1, 3, 5-trihydroxybenzene) affects motor functional deficits in a unilaterally 6-hydroxydopamine (6-OHDA)-injected rat PD model. 6-OHDA is one of the most commonly used neurotoxins in PD models [4], [5]. Because it shares a similarity with the endogenous neurotransmitter dopamine, 6-OHDA can enter dopaminergic neurons via the dopamine transporter. 6-OHDA is known to damage dopaminergic neurons by inhibiting the activities of the complex 1 enzyme in mitochondria and thus induces oxidative stress within neurons [4],[5]. As catecholamines are generally unable to penetrate the developed blood brain barrier [5], 6-OHDA is usually administered directly into the striatum or medial forebrain bundle in mice or rats via stereotaxic procedures [6].

Phloroglucinol is a member of the polyphenol of organic chemical group and is one of the phlorotannin components that is abundantly found in *Ecklonia cava* (edible brown algae), which belongs to the Laminariaceae family. Several published reports have described the protective effects of phloroglucinol against H_2_O_2_-induced oxidative stress in *in vitro* and *in vivo* [7], [8].

Here, we demonstrate that phloroglucinol administration attenuated motor functional deficits evaluated with the rota-rod and apomorphine-induced rotation tests in a unilateral 6-OHDA lesioned rat PD model. Phloroglucinol was shown to ameliorate the loss of dopaminergic neurons as assessed with tyrosine hydroxylase (TH) protein levels. Moreover, phloroglucinol administration rescued the reduction in synapse formation as evaluated with the protein levels of synaptophysin, a presynaptic marker for a functional synapse, in the midbrain of 6-OHDA-injected rats.

In SH-SY5Y cultures, cytotoxicity of 6-OHDA was reduced by phloroglucinol pre-treatment. In addition, an increase in reactive oxygen species (ROS) and in lipid peroxidation, protein carbonyl formation and DNA oxidation induced by 6-OHDA treatment, was ameliorated by phloroglucinol in the cells. Furthermore, phloroglucinol treatment rescued the reduction of nuclear factor-erythroid-2 p45-related factor (Nrf2) in nuclear fraction, the downstream molecules of Nrf2, i.e., catalase and glutathione peroxidase protein levels caused by 6-OHDA. Taken together, phloroglucinol has a therapeutic potential for treatment of PD, potentially due to its ROS-scavenging and antioxidant enzyme-upregulating properties.

## Materials and Methods

### Reagents

Phloroglucinol, 6-OHDA, MTT and Hoechst 33342 dye were purchased from Sigma Chemical Company (St. Louis, MO, USA). 2′, 7′-dichlorofluorescein diacetate (DCF-DA) was, from Invitrogen (Carlsbad, CA, USA). Primary antibodies for catalase, glutathione peroxidase, Akt, p-Akt, p-Nrf2 and TH were purchased from Santa Cruz Biotechnology (Santa Cruz, CA, USA). The anti-synaptophysin monoclonal antibody was, from Millipore (Billerica, MA, USA).

### Animals

In this study, 7-week-old male Sprague Dawley rats (200–250 g) were used (Central Laboratory Animal Incorporation, Seoul, Korea). The rats were housed in groups of two per cage with *ad libitum* access to food and water and were maintained under a controlled temperature (22±2°C), relative humidity (50±10%) and a 12 h light/dark cycle (light on at 8:00 AM). All animal experiments were performed according to guidelines that were approved by the Animal Experiments of Ethics Committee of Seoul National University. This study was also approved by Animal Experiments of Ethics Committee of Seoul National University.

### Animal surgery

Animals were randomly divided into four groups (n = 7 per group). The first group consisted of control animals that were administered PBS into the right medial forebrain bundle. The second group consisted of animals administered with 6 µmole of phloroglucinol into right medial forebrain bundle. The third group consisted of 6-OHDA-lesioned animals that were administered 18 µg of 6-OHDA into the right medial forebrain bundle. The fourth group consisted of animals administered with 18 µg of 6-OHDA and 6 µmole of phloroglucinol into right medial forebrain bundle. Animals were anesthetized with sodium pentobarbital (3 ml/kg, i.p., Entobar®, Hanlim Pharm. Co., Korea) and placed on a Kopf stereotaxic frame (David Kopf Instruments, CA, USA).The animals were implanted with a guide cannula (26 gauge syringe, Hamilton, Nevada, USA) into the right medial forebrain bundle using the following coordinate from bregma: −4.4 anterior/posterior (AP), −1.1medial/lateral(ML), −0.8 dorsal/ventral (DV) from the dura, at a rate of 0.3 µl/min.

### Rota-rod test

The rota-rod test was performed 2 weeks after surgery and was performed as described previously [9]. Animals were placed on the rota-rod treadmill (UgoBasile, Italy) at an accelerating speed from 6 round/min to 30 round/min for 3 min. The latency to fall was measured and three training sessions were given before experiment. The animals performed seven trials in a day.

### Apomorphine-induced rotation test

The apomorphine-induced rotation test was performed 3 weeks after surgery. Apomorphine was subcutaneously injected at a dose of 0.5 mg/kg (Sigma) and the rotation was monitored for 60 min using the apparatus described by Ungerstedt and Arbuthnott [9]. The results were expressed as the contralateral or ipsilateral net turns/60 min.

### Perfusion and tissue preparation

For tissue preparation, rats were deeply anesthetized with sodium pentobarbital (60 mg/kg i.p.). They were perfused transcardially with heparin dissolved in PBS (pH. 7.2). The brains from three animals were dissected and fixed in 4% paraformaldehyde in PBS (pH 7.2) prior to make paraffin-embedded sectioning. The other four animals from each group were sacrificed using similar perfusion method and the brains were dissected. The dissected brain tissues were frozen at −80°C for Western blotting.

### Western blotting

SH-SY5Y cells or brain tissues were lysed in a lysis buffer containing 50 mM Tris, pH 7.4, 150 mM NaCl, 1% Triton, 0.5% SDS, 0.5% sodium deoxycholate, and protease inhibitors. Proteins were resolved on an SDS polyacrylamide gel, electrophoresed with 30 to 50 µg of protein/lane, and transferred onto a nitrocellulose membrane (GE Healthcare, Little Chalfont, Buckinghamshire, UK). Subsequently, the blot was incubated with primary antibodies. Immunoreactive bands were detected using a horseradish peroxidase-conjugated secondary antibody (GE Healthcare Pharmacia, Uppsala, Sweden) and visualized using an enhanced chemiluminescence detection system (GE Healthcare AB, Stockholm, Sweden).

### Immunohistochemistry (IHC)

Brains were cut into 4 µm-thick sagittal sections. For IHC with antibodies against TH and synaptophysin, paraffin slides were de-paraffinized and hydrated in a graded series of ethanol solutions of decreasing concentrations (100%-70% ethanol). Next, the slides were washed with 0.1 M Tris-buffered saline (TBS, pH.7.8) and the antigen retrieval step was performed in citric acid buffer (0.01 M, pH.6.0) for 10 min at 56°C. Then, 1% H_2_O_2_ in methanol was applied for 30 min to remove the endogenous peroxide in the tissue. The slides were subsequently incubated with blocking buffer (10% BSA and 0.2% Triton X-100 in 0.1 M TBS) for 1 h at room temperature and then with the primary antibodies, rabbit polyclonal anti-TH (1∶200) and mouse monoclonal anti-synaptophysin (1∶2000) diluted in blocking buffer overnight at 4°C. Next, the slides were washed with 0.2% Triton X-100 in 0.1 M TBS and secondary antibodies were added on to the slides for 1.5 h at room temperature. The secondary antibodies used were Alexa Fluor® 488 goat anti-mouse IgG (1∶200, Invitrogen, CA, USA) and Alexa Fluor® 555 goat anti-rabbit IgG (1∶4000, Invitrogen). DAPI was in turn incubated for 5 min for the visualization of nuclei. The slides were washed with 0.2% Triton X-100 in 0.1 M TBS and then mounted with FluorSave™ Reagent (Calbiochem; La Jolla, CA, USA). All of the slides were examined on an LSM510 confocal microscope (Zeiss, Jena, Germany). The number of TH-positive dopaminergic neurons was quantified by counting in each of the stereotaxic regions of the *Substantia nigra* in both the contralateral and ipsilateral hemispheres of the brain.

### Cell cultures

SH-SY5Y cells were obtained from the American Type Culture Collection (Rockville, MD, USA), and the cells were maintained at 37°C in an incubator with a humidified atmosphere of 5% CO_2_ and cultured in Dulbecco's modified Eagle's medium containing 10% heat-inactivated fetal calf serum, streptomycin (100 µg/ml) and penicillin (100 units/ml).

### Intracellular ROS measurements

The DCF-DA method was used to detect the level of intracellular ROS [10]. Cells were seeded in a 96-well plate at 1.5×10^4^ cells/well and 16 h after plating, the cells were treated with phloroglucinol at 10 µg/ml. After 1 h, 90 µM of 6-OHDA was added to the plates. The cells were incubated for an additional 24 h at 37°C. After the addition of 25 µM of DCF-DA solution for 10 min, the fluorescence of DCF was detected using a PerkinElmer LS-5B spectrofluorometer and flow cytometry (Becton Dickinson, Mountain View, CA, USA). In addition, microscopic images were collected using a Laser Scanning Microscope 5 PASCAL (Carl Zeiss, Jena, Germany).

### Lipid peroxidation assay

Lipid peroxidation was assayed by the determination of 8-isoprostanelevels [11]. The levels of 8-isoprostane were measured in the culture medium by a commercial enzyme immunoassay (Cayman Chemical, Ann Arbor, MI, USA), which was performed according to the manufacturer's instructions.

### Protein carbonylation assay

The amount of carbonyl formation in protein was determined using an Oxiselect™ protein carbonyl enzyme-linked immunosorbent assay kit purchased from Cell Biolabs (San Diego, CA, USA), according to the manufacturer's instruction.

### 8-hydroxyguanine detection

The 8-hydroxyguanine content in DNA was determined using a Bioxytech 8-OHdG-ELISA kit purchased from OXIS Health Products (Portland, OR, USA) and performed according to the manufacturer's instructions. Cellular DNA was isolated using the DNAzol reagent (Life Technologies, Grand Island, NY, USA) and quantified using a spectrophotometer.

### Preparation of nuclear extracts

Cells were harvested and were subsequently lysed on ice with 1 ml of lysis buffer (10 mM Tris–HCl, pH 7.9, 10 mM NaCl, 3 mM MgCl_2_ and 1% NP-40) for 4 min. After centrifugation for 10 min at 3000× g, the pellets were re-suspended in 50 µl of extraction buffer (20 mM HEPES, pH 7.9, 20% glycerol, 1.5 mM MgCl_2_, 0.2 mM EDTA, 1 mM DTT and 1 mM PMSF), incubated on ice for 30 min and were centrifuged at 13,000× g for 5 min. Supernatants (nuclear protein) were stored at −70°C after determination of the protein concentration.

### Cell viability measurements

The effects of 6-OHDA on cell viability and phloroglucinol against 6-OHDA-induced cell death were determined by an MTT assay, which is based on the cleavage of a tetrazolium salt by mitochondrial dehydrogenase in viable cells [12]. Cells were seeded in a 96-well plate at a concentration of 1×10^5^ cells/ml, and 6-OHDA at 25, 50, 100, and 200 µM was added to the plate and incubated for an additional 48 h at 37°C to determine cytotoxicity. To evaluate the protective effects of phloroglucinol against 6-OHDA toxicity, cells were pre-incubated with different concentrations of phloroglucinol for 1 h and then treated with 90 µM of 6-OHDA for an additional 48 h. Subsequently, 50 µl of MTT stock solution (2 mg/ml) was added to each well for a total reaction volume of 200 µl. After incubating for 4 h, the plate was centrifuged at 800× g for 5 min and the supernatants were aspirated. The formazan crystals in each well were dissolved in 150 µl of DMSO and the absorbance (540 nm) was read on a scanning multi-well spectrophotometer.

### Nuclear staining with Hoechst 33342

Cells were pre-treated with phloroglucinol for 1 h and 90 µM of 6-OHDA was then treated and incubated for additional 24 h at 37°C. Next, 20 µM of Hoechst 33342, a DNA-specific fluorescent dye, was added to each well and the plates were incubated for 10 min at 37°C. The degree of nuclear condensation was evaluated in the stained cells under a fluorescence microscope equipped with a Cool SNAP-Pro color digital camera (Media Cybernetics, Meyer, Housten, TX, USA).

### Catalase activity assay

50 µg of protein was added to 50 mM phosphate buffer (pH 7) containing 100 mM H_2_O_2_. The reaction mixture was incubated for 2 min at 37°C and the absorbance was monitored at 240 nm for 5 min. The change in absorbance with time was proportional to the breakdown of H_2_O_2_. The catalase activity was expressed as units/mg protein and one unit of enzyme activity was defined as the amount of enzyme required to breakdown 1 µM of H_2_O_2_.

### Glutathione peroxidase activity assay

Harvested cells were suspended in 10 mM phosphate buffer (pH 7.5). The cells were centrifuged at 12,000× g for 30 min at 4°C to remove the tissue debris. Glutathione peroxidase activity was determined using the FR 17 assay kit (Oxford Biomedical Research, MI, USA), which was performed according to the manufacturer's protocol. The enzyme reaction was assessed by adding the substrate, tert-butyl hydroperoxide, and was measured at an absorbance of 340 nm. The rate at which the absorbance decreases was directly proportional to the activity of the glutathione peroxidase (expressed as mU/ml).

### Transient transfection of ARE promoter and luciferase assay

A day before transfection, cells were sub-cultured at a density of 1×10^6^ cells in a 60 mm dish to reach approximately 60–80% confluence. The cells were transiently transfected with a plasmid harboring the ARE-luciferase gene using the transfection reagent lipofectamine 2000, according to the manufacturer's instructions (Invitrogen). After overnight transfection, cells were treated with 10 µg/ml of phloroglucinol and 1 h after 6-OHDA treatment. Cells were then washed twice with PBS and lysed with reporter lysis buffer. After vortex-mixing and centrifugation at 12,000× g for 1 min at 4°C, the supernatant was stored at −70°C for the luciferase assay. After 20 µl of the cell extract was mixed with 100 µl of the luciferase assay reagent at room temperature, the mixture was placed in a luminometer in order to measure the light produced.

### Statistics

All of the data are presented as the mean ± SEM. Statistical significance was tested using one-way ANOVA followed by Duncan *post hoc* tests using PASW statistics 18 (SPSS Inc. Chicago, IL, USA). The results were considered to be statistically significant if the value was <0.05.

## Results

### Phloroglucinol ameliorates 6-OHDA-induced motor functional deficits in rats

**Figure 1:**
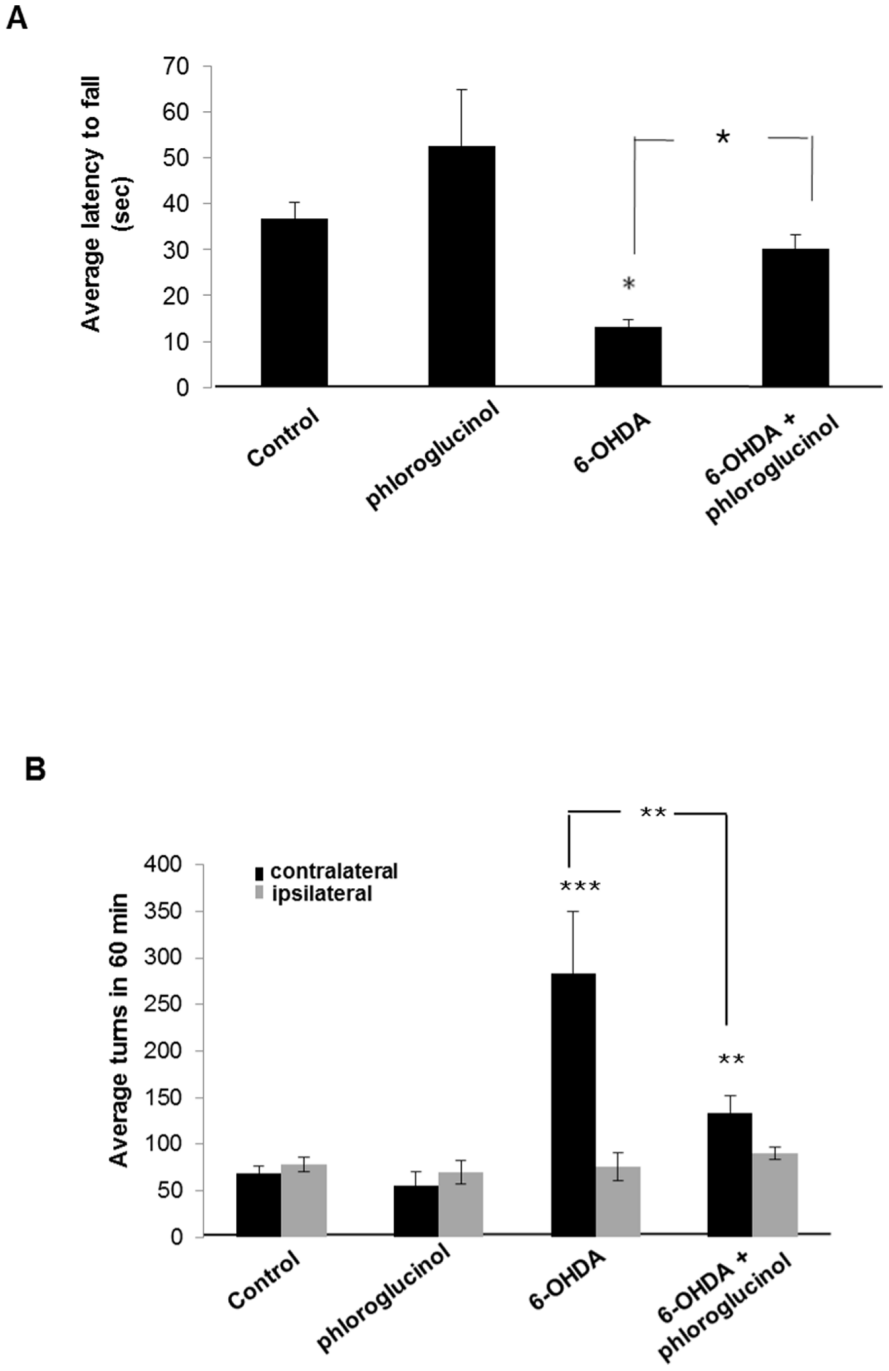
Phloroglucinol ameliorates 6-OHDA induced motor deficits in rats. (**A**) All of the animals of the four groups performed seven trials of the accelerated rota-rod test at 2 weeks after administration of vehicle, phloroglucinol, 6-OHDA or 6-OHDA plus phloroglucinol. Animals were placed on the rota-rod treadmill at an accelerating speed from 6 round/min to 30 round/min in 3 min. The latency to fall was measured and three training sessions were performed before each test. Animals were tested with seven trials on a given day. (ANOVA, *post-hoc* by Duncan, * *p*<0.05). (**B**) All of the animals of the four groups performed the apomorphine-induced rotation test at 3 weeks after the administration of vehicle, phloroglucinol, 6-OHDA or 6-OHDA plus phloroglucinol. Apomorphine was subcutaneously injected at 0.5 mg/kg, and the rotation was monitored for 60 min using the apparatus described by Ungerstedt and Arbuthnott [9]. The results were expressed as contralateral or ipsilateral net turns/60 min. (ANOVA, *post-hoc* by Duncan, ** *p*<0.01, ***p<0.001).

To evaluate motor coordination and balance, each group of rats was analyzed by rota-rod tests 2 weeks after 6-OHDA injections into the right medial forebrain bundle. The rota-rod task is a performance test based on a rotating rod with forced motor activity being applied, usually by a rodent. At an accelerating speed, the 6-OHDA-lesioned group exhibited highly impaired motor function compared to the vehicle, phosphate buffered saline (PBS)-treated group (rota-rod: 13.2±1.6 sec and 36.9±3.5 sec, respectively, *p*<0.05). However, rats that were co-administered with phloroglucinol and 6-OHDA (rota-rod: 30.1±3.0 sec) showed improved motor function compared with the 6-OHDA-lesioned group (*p*<0.05) and reached significant recovery at similar levels to the control group, although the administration of phloroglucinol alone was not found to affect the motor function assessed with rota-rod (Figure 1A).

We also performed apomorphine-induced rotation tests. Because the deficiency of dopamine leads to the supersensitivity of dopamine receptors in the striatum, apomorphine is commonly used to evaluate the degree of dopaminergic neuronal degeneration as it is a non-selective dopamine receptor agonist [5], [13], [14]. In the apomorphine-induced rotation test, the rats exhibited rotational behavior in the direction opposite to the side of the lesion. To examine the effects of phloroglucinol in 6-OHDA lesioned rats, apomorphine (0.5 mg/kg) was subcutaneously administrated into the rats. Rats with a unilateral infusion of 6-OHDA into the right medial forebrain bundle exhibited increased apomorphine-induced contraversive (left) rotations: 282.9±66.8 turns/h compared to PBS-infused rats: 69.0±7.8 turns/h (***p<0.001, Figure 1B). With phloroglucinol co-administration, the number of contraversive rotations in the 6-OHDA-lesioned rats was significantly reduced compared with the lesioned group co-administered with vehicle (134.0±18.4 turns/h, **p<0.01), while the administration of phloroglucinol alone did not show any difference in contraversive rotations after apomorphine injection (56.1±13.9 turns/h), compared to PBS-infused rats (69.0±7.8 turns/h).

However, changes in the number of ipsiversive rotations were not observed in any of the four groups. These results indicated that administration of phloroglucinol protected against the 6-OHDA-induced motor functional impairments in rats.

### Phloroglucinol attenuates 6-OHDA-induced loss of dopaminergic neurons in the midbrain

**Figure 2:**
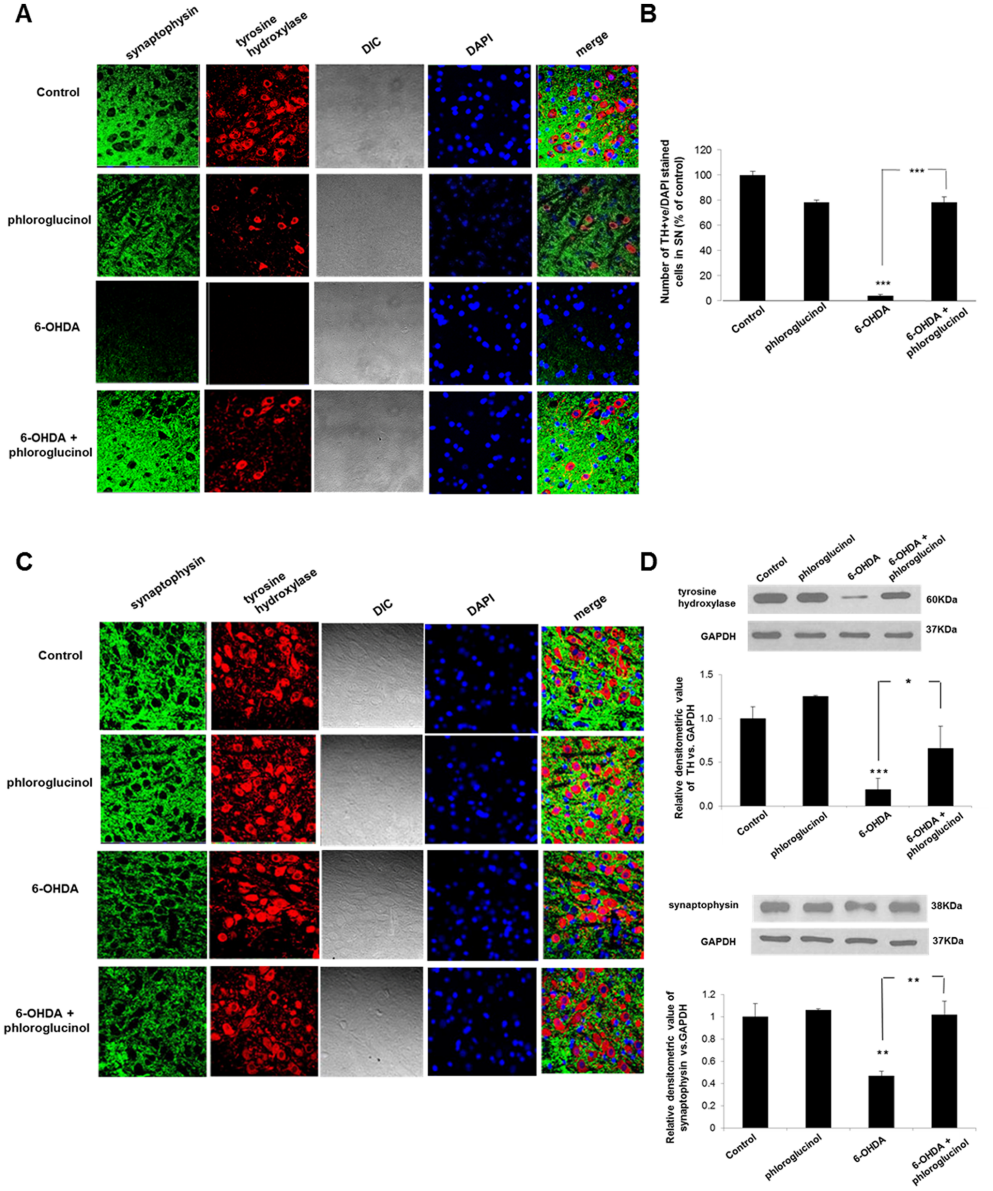
Phloroglucinol attenuates 6-OHDA-induced loss of dopaminergic neurons and synapses in the midbrain. (**A**) (**C**) Immunoreactivity of TH (red), synaptophysin (green) and DAPI (blue) was evaluated in ipsilateral (A) and contralateral sides (C) of the midbrain of each animal group by IHC, resspectively. All of the slides were examined on a LSM510 confocal microscope. (**B**) Quantitative analysis of TH immunoreactivity was performed and marked as a percentage of the control group. (**D**) The protein levels of TH and synaptophysin in the midbrain region were assessed with Western blotting. The relative densitometric value of TH or synaptophysin *vs.* that of glyceraldehyde-3-phosphate dehydrogenase (GAPDH) is shown in graph. (ANOVA, post-hoc by Duncan,*p<0.05, **p<0.01, *** *p*<0.001).

To discern the neuroprotective effects of phloroglucinol on dopaminergic neurons in the midbrain, we checked the protein levels and immunoreactivities of TH, a marker for dopaminergic neurons by Western blotting and IHC, respectively. The unilateral infusion of 6-OHDA produced a significant reduction in the percentage of TH-positive neurons vs. DAPI (4′, 6-diamidino-2-phenylindole, dihydrochloride, Invitrogen) stained cells in the *Substantia nigra* (3.9±2.9%, *p*<0.001) compared to the control group (Figures 2A, B). However, the co-administration of 6-OHDA and phloroglucinol significantly restored the loss of TH positive neurons in the brain region (79.4±18.6%, Figures 2A, B), although the administration of phloroglucinol alone did not affect the loss of dopaminergic neurons assessed by TH immunoreactivity (78.3±2.0%, Figures 2A, B). Western blot results also showed that the protein levels of TH in the midbrain was remarkably reduced in 6-OHDA injected mice, compared to the control group (Figure 2D). However, the co-administration of 6-OHDA and phloroglucinol significantly restored the loss of TH protein levels in the brain region, although the administration of phloroglucinol alone did not affect the TH protein levels (Figure 2D). This result shows that phloroglucinol protects dopaminergic neurons from 6-OHDA-induced neurodegeneration and is consistent with the results of the recovery of motor deficits that are shown Figures 1A and B.

### Phloroglucinol rescues 6-OHDA-induced reductions in the synapses between dopaminergic neurons in the midbrain

Synaptophysin is a presynaptic marker for functional synapses. We checked the protein levels and immunoreactivities of synaptophysin by Western blotting and IHC, respectively. The results showed a considerable loss of the expression of synaptophysin in sagittal sections of the midbrain, which was associated with TH immunoreactivity (Figure 2A). Phloroglucinol co-administration restored the reduction in synaptophysin immunoreactivity demonstrated in 6-OHDA-treated animals (Figure 2A). However, there were no differences detected between the immunoreactivities against TH and synaptophysin in the contralateral side of the brain among all of the animal groups (Figure 2C).

Western blot results also showed the protein levels of synaptophysin in the midbrain was significantly reduced in 6-OHDA injected mice, compared to the control group (Figure 2D). However, the co-administration of 6-OHDA and phloroglucinol almost completely restored the loss of synaptophysin protein levels in the brain region, although the administration of phloroglucinol alone did not affect the synaptophysin protein levels (Figure 2D). The results shown in Figure 2 indicate that phloroglucinol attenuated the 6-OHDA-induced loss of TH- positive dopaminergic neurons and of synapses formed between the neurons in the midbrain. The subtle discrepancy between the protein levels and immunoreactivity of TH or synaptophysin in Western blot and IHC is possibly due to the fact that Western blotting was performed with the total midbrain tissues.

### Phloroglucinol exerts protective effects against 6-OHDA-induced damage in SH-SY5Y cells

**Figure 3:**
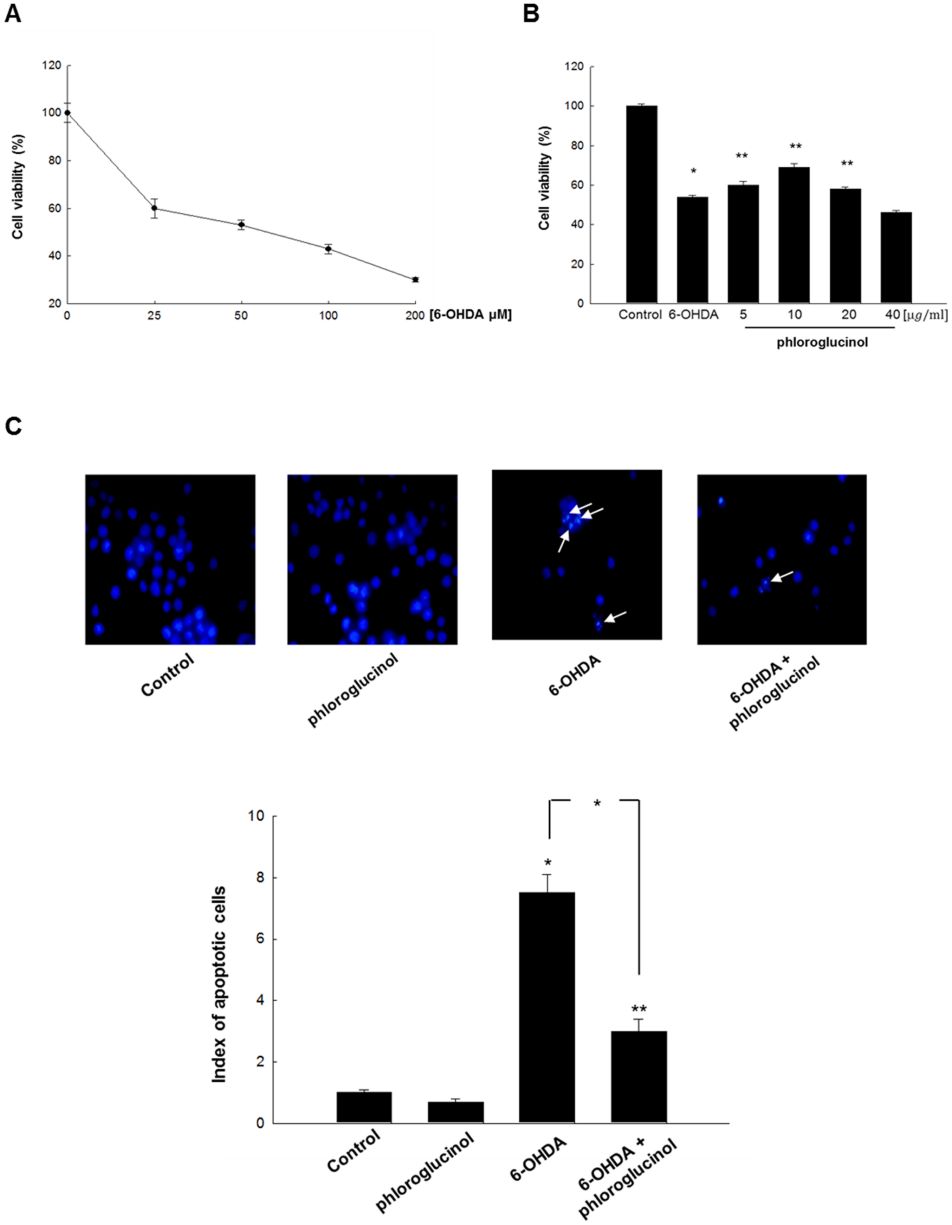
Phloroglucinol exerts protective effects against 6-OHDA in SH-SY5Y cells. (**A**) Cell viability was measured by an MTT assay. SH-SY5Y cells were treated with a range of different concentrations of 6-OHDA (0, 25, 50, 100, and 200 µM of 6-OHDA). The IC_50_ was determined to be 90 µM 6-OHDA. (**B**) The cells were pre-treated with phloroglucinol (5, 10, 20, and 40 µg/ml) 1 h before treatment with 6-OHDA. The percentage of cell viability was measured compared to control. (ANOVA, *post-hoc* by Duncan, * *p*<0.05, **p<0.01). (**C**) Apoptotic bodies (arrows) were observed in cells stained with Hoechst 33342 dye and quantified by fluorescence microscopy. (ANOVA, *post-hoc* by Duncan, * *p*<0.05, **p<0.01).

We examined the neuroprotective effects and its mechanisms in *in vitro* by using SH-SY5Y cells, the neuroblastoma cell line. First, we confirmed the cytotoxic effects of 6-OHDA in the cells, by performing an 3-(4, 5-dimethylthiazol-2-yl)-2, 5-diphenyltetrazolium] bromide 3-(4, 5-dimethylthiazol-2-yl)-2, 5-diphenyltetrazolium] bromide (MTT) assay. Treatment with 6-OHDA at 90 µM for 48 h showed 50% of cytotoxicity in the cells (Figure 3A). Based on these results, we used a concentration of 90 µM of 6-OHDA for subsequent experiments. We found that phloroglucinol pre-treatment for 1 h prior to 6-OHDA treatment for 48 h significantly attenuated the cytotoxic effects induced by 6-OHDA in cells treated with 5, 10, 20 µg/ml phloroglucinol (Figure 3B). Among these concentrations, treatment with 10 µg/ml of phloroglucinol showed the highest cell viability (68.6±0.4% vs. negative control). Therefore, we used the concentration of 10 µg/ml phloroglucinol for subsequent experiments.

Previous studies showed that 6-OHDA induces apoptosis [15], [16], as evidenced by the presence of apoptotic bodies. Here, we found that nuclear fragmentation induced by 6-OHDA treatment was dramatically reduced when the cells were pre-treated with phloroglucinol for 1 h, prior to 6-OHDA treatment, with a significant reduction in the apoptotic index from 7.5 to 3 (*p<0.05, Figure 3C).

### Phloroglucinol prevents the increase in intracellular ROS caused by 6-OHDA treatment in SH-SY5Y cells

**Figure 4:**
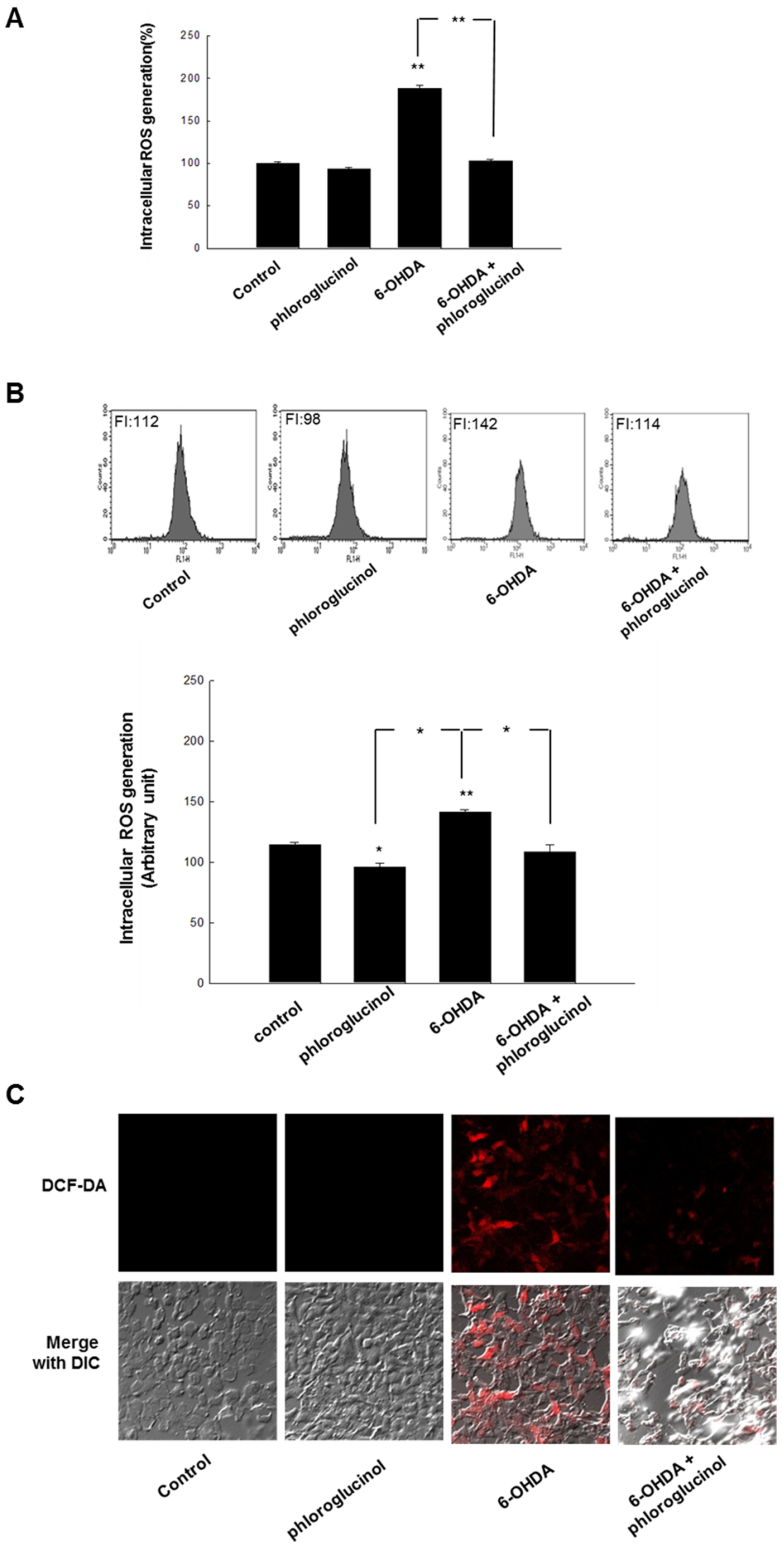
Phloroglucinol reduces the intracellular ROS caused by treatment with 6-OHDA in SH-SY5Y cells. (**A**) Cells were seeded in a 96-well plate at 1.5×10^4^ cells/well. After16 h plating, the cells were treated with phloroglucinol at 10 µg/ml. After 1 h, 90 µM of 6-OHDA was added to the plates. The cells were incubated for an additional 24 h at 37°C. After the addition of 25 µM of DCF-DA solution for 10 min, the fluorescence of the DCF was detected using a PerkinElmer LS-5B spectrofluorometer. (**B**) The intracellular ROS level was evaluated using a flow cytometry as previously described. (**C**) Microscopic images were collected using laser scanning confocal microscope 5 PASCAL. (ANOVA, *post-hoc* by Duncan, * *p*<0.05, **p<0.01).

6-OHDA is a well-known neurotoxin that mimics the Parkisonian state *in vitro* and *in vivo*. It interferes with the normal oxidative phosphorylation process that occurs in mitochondria, resulting in the formation of free radicals. We evaluated whether phloroglucinol has effects against the oxidative stress exerted by 6-OHDA treatment for 48 h. We found that phloroglucinol pre-treatment for 1 h prior to 6-OHDA treatment significantly attenuated the increase in intracellular ROS (Figures 4A, B and C). Increased ROS levels returned to levels nearly similar to those of the control group with pre-treatment of phloroglucinol for 1 h, which is detected by using spectrofluometer, flow cytometry, and confocal microscopy, respectively, followed by DCF-DA staining. Representative figures for the fluorescence of DCF-DA are shown in Figure 4C.

### Phloroglucinol down-regulates lipid peroxidation, protein carbonylation and DNA base modification induced by 6-OHDA treatment in SH-SY5Y cells

**Figure 5:**
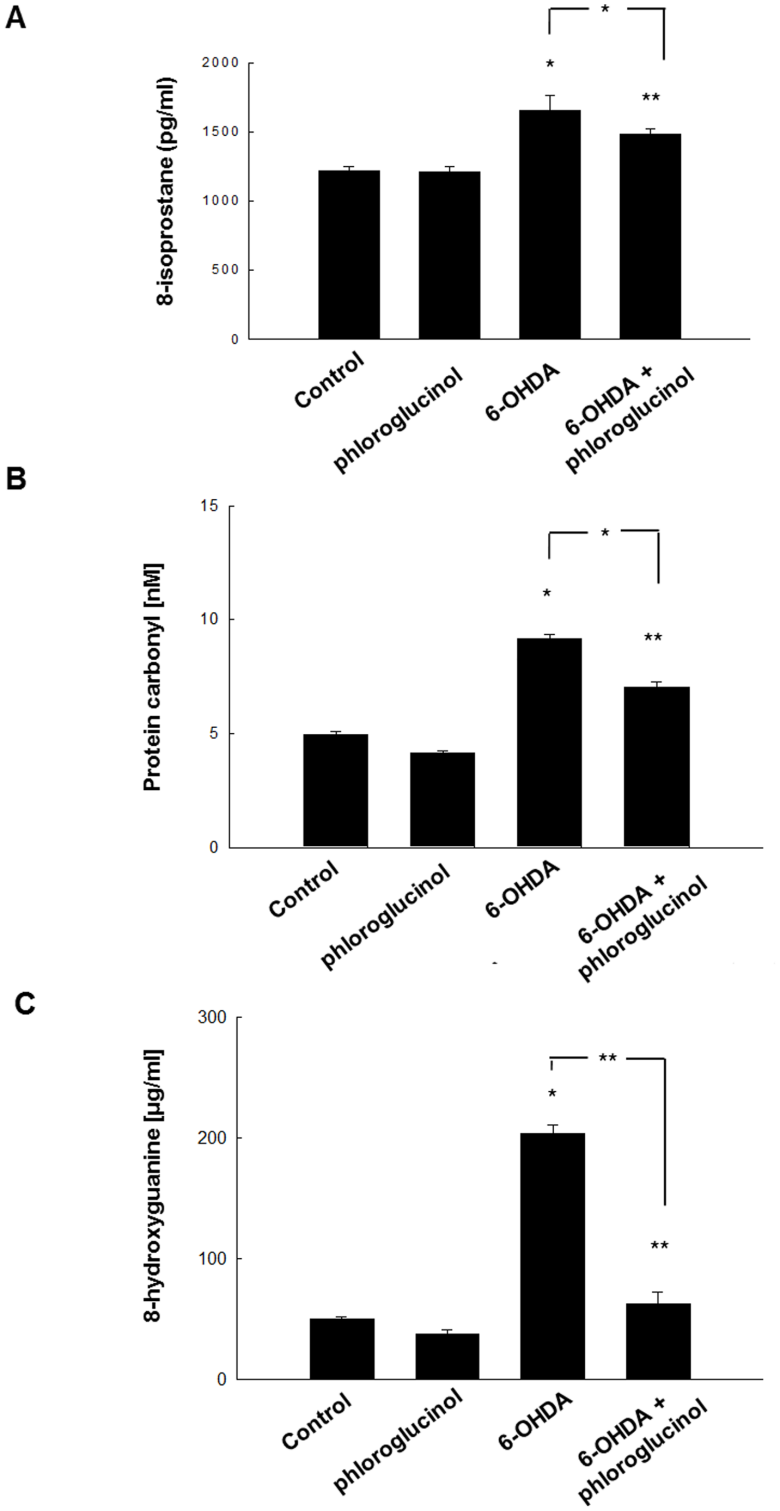
Phloroglucinol down-regulates lipid peroxidation, protein carbonylation and DNA base modification induced by 6-OHDA treatment in SH-SY5Y cells. (**A**) Lipid peroxidation was assayed by determination of 8-isoprostane levels. 8-Isoprostane levels were determined in the culture medium by use of a commercial enzyme immunoassay and were performed according to the manufacturer's instructions. (**B**) The amount of carbonyl formation in protein was determined using an ELISA kit and expressed as nM. (**C**) The 8-hydroxyguanine content in DNA was determined using a Bioxytech 8-OHdG-ELISA kit purchased from OXIS Health Products and was performed according to the manufacturer's instructions. Cellular DNA was isolated using the DNAzol reagent and quantified using a spectrophotometer. (ANOVA, *post-hoc* by Duncan, * *p*<0.05, **p<0.01).

Increased ROS levels are known to induce lipid peroxidation, protein carbonylation and DNA oxidation. Here, we investigated whether phloroglucinol affects lipid peroxidation (8-isoprostane), protein carbonylation and DNA base modification (8-hydroxyguanine). We found that 8-isoprostane contents were reduced from 1651±110 pg/ml to 1482±41 pg/ml by pre-treatment with phloroglucinol for 1 h (Figure 5A).

Protein carbonyl concentrations were significantly down-regulated from 9.2±0.2 to 7.0±0.3 nM by pre-treatment with phloroglucinol for 1 h prior to 6-OHDA treatment (Figure 5B). Together with lipid peroxidation and protein carbonylation, the contents of 8-hydroxyguanine, which signifies DNA base modification, are well correlated with oxidative stress [17]. We found that phloroglucinol pre-treatment significantly attenuated the increase in 8-hydroxyguanine induced by 6-OHDA treatment for 48 h (Figure 5C).

### Phloroglucinol attenuates the 6-OHDA-mediated loss of the activity and expression of antioxidant enzymes

**Figure 6:**
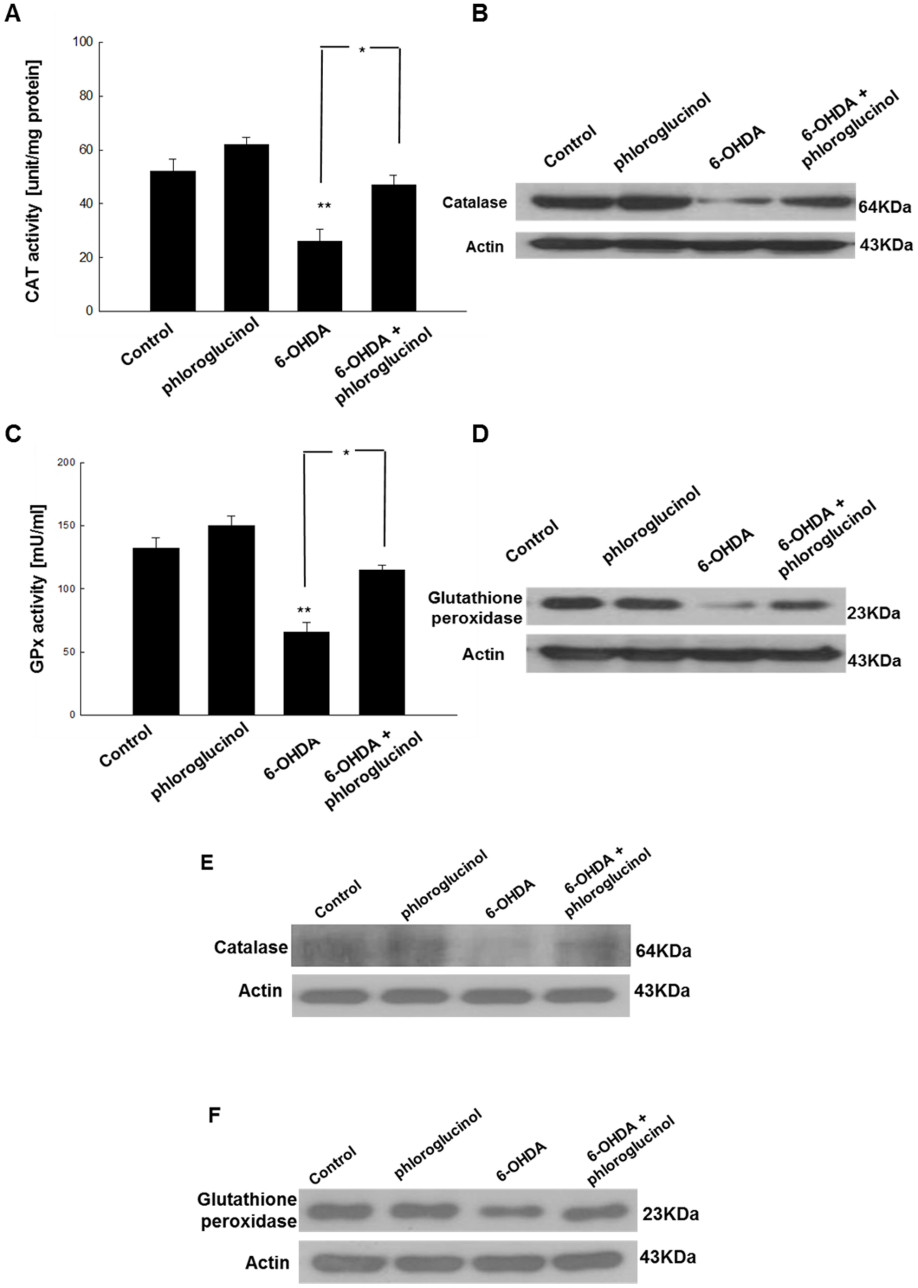
Phloroglucinol attenuates the 6-OHDA-mediated loss of antioxidant enzymes in SH-SY5Y cells and rat brains. (**A**) 50 µg of protein was added to 50 mM phosphate buffer (pH 7) containing 100 mM H_2_O_2_. The reaction mixture was incubated for 2 min at 37°C and the absorbance was monitored at 240 nm for 5 min. The change in absorbance over time was proportional to the breakdown of H_2_O_2_. The catalase activity was expressed as units/mg protein and one unit of enzyme activity was defined as the amount of enzyme required to breakdown 1 µM of H_2_O_2_. (**B**) Protein levels of catalase were evaluated with Western blotting in SH-SY5Y cells. (**C**) The harvested cells were suspended in 10 mM phosphate buffer (pH 7.5). The cells were centrifuged at 12,000× g for 30 min at 4°C to remove the tissue debris. Glutathione peroxidase activity was determined using the FR 17 assay kit according to the manufacturer's protocol. The enzyme reaction was assessed by adding the substrate, tert-butyl hydroperoxide and was recorded at 340 nm. The rate at which the absorbance (340 nm) decreased is directly proportional to the activity of glutathione peroxidase (expressed in mU/ml). (**D**) The protein levels of glutathione peroxidase in SH-SY5Y cells were evaluated with Western blotting in SH-SY5Y cells. (**E**) The protein levels of catalase in the ipsilateral midbrain region were assessed with Western blotting. (**F**) The protein levels of glutathione peroxidase in the ipsilateral midbrain region were assessed with Western blotting. (ANOVA, *post-hoc* by Duncan, * *p*<0.05, **p<0.01).

Catalase and glutathione peroxidase are vital antioxidant enzymes that protect cells against oxidative damage. To investigate whether phloroglucinol reduces the increase in ROS induced by 6-OHDA treatment through the changes in antioxidant enzymes, the activities and protein expression of catalase and glutathione peroxidase in phloroglucinol-treated cells were measured. 6-OHDA treatment significantly decreased catalase activity to 25.9±4.6 U/mg protein (*p*<0.01); however, pre-treatment with phloroglucinol in 6-OHDA-treated cells restored catalase activity to 46.9±3.7 U/mg protein (*p*<0.05, Figure 6A). With respect to the glutathione peroxidase, the exposure of SH-SY5Y cells to 6-OHDA significantly decreased the glutathione peroxidase activity to 66.3±7.7 mU/ml protein and pre-treatment with phloroglucinol restored the activity to 114.5±2.1 mU/ml in 6-OHDA-treated cells (*p*<0.05, Figure 6C).

In addition, 6-OHDA treatment decreased the protein level of antioxidant enzymes, whereas the pre-treatment with phloroglucinol in 6-OHDA-treated cells reversed this effect (Figures 6B, D). These results are consistent with our previous report that phloroglucinol up-regulates the activity and the protein level of antioxidant enzymes such as catalase and glutathione peroxidase [7].

Taken together, these results indicate that phloroglucinol can increase both the activities and the protein amount of antioxidant enzymes such as catalase and glutathione peroxidase, which were reduced in 6-OHDA-treated cells.

In the midbrains of control, phloroglucinol, 6-OHDA or 6-OHDA plus phloroglucinol administered rats, the administration of 6-OHDA significantly down-regulated the protein level of catalase, compared with the control group. However, the co-administration of 6-OHDA and phloroglucinol reversed the reduction in the protein level of catalase (Figure 6E). We also checked the protein level of glutathione peroxidase. As with catalase, the administration of 6-OHDA significantly down-regulated the protein level of glutathione peroxidase, compared with the control group. However, the co-administration of 6-OHDA and phloroglucinol restored the reduction in the protein level of glutathione peroxidase (Figure 6F).

### Phloroglucinol restores the decrease in nuclear Nrf2 induced by 6-OHDA treatment

Nrf2 is a redox-sensitive transcription factor that positively regulates the expression of genes encoding antioxidants, xenobiotic detoxification enzymes, and drug efflux pumps [14], [15]. Under normal physiologic conditions, Nrf2 is sequestered in the cytoplasm, whereas upon stimulation, Nrf2 or p-Nrf2 is translocated into the nucleus and activates the transcription of genes containing an antioxidant response element (ARE) in the promoter region [18], [19], [20].

**Figure 7:**
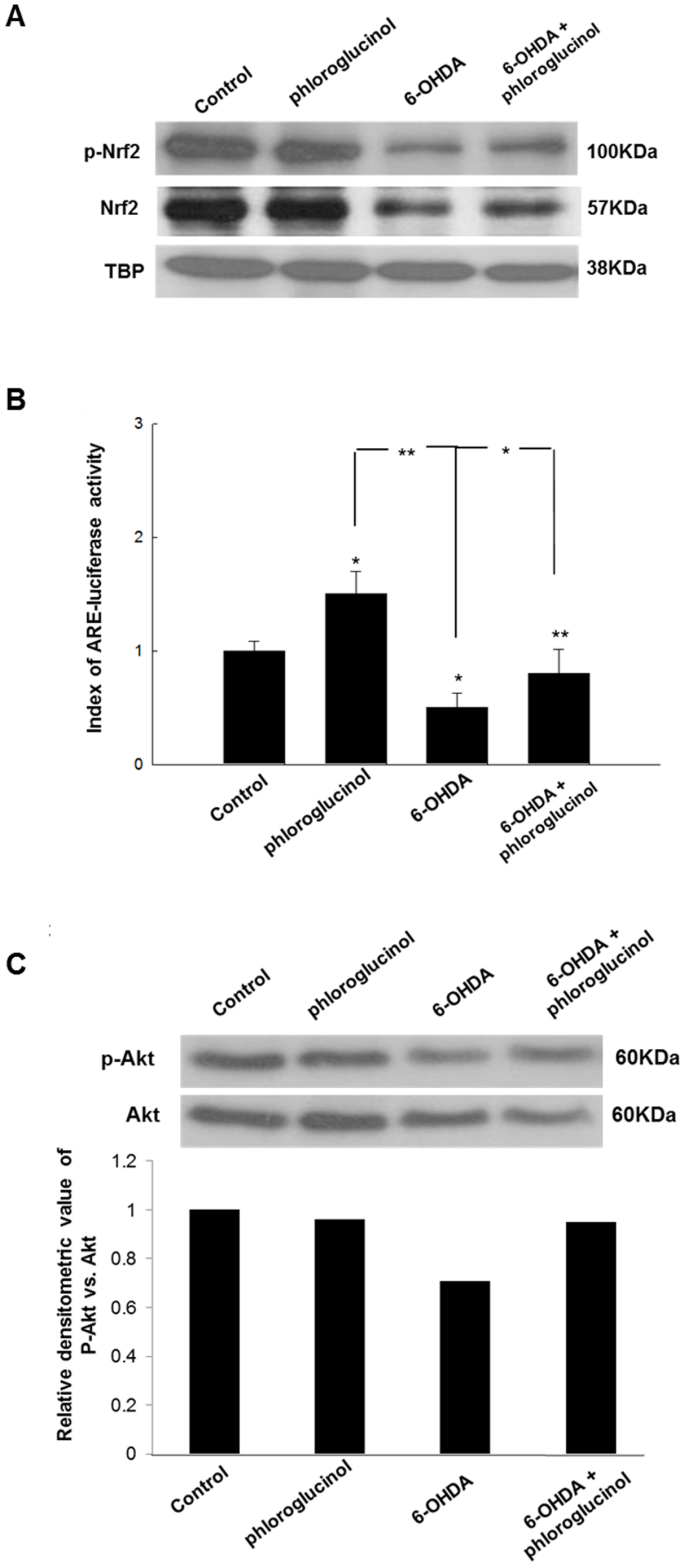
Phloroglucinol attenuates the 6-OHDA-mediated loss of Nrf2 and p-Nrf2 in the nuclear fraction and p-Akt in SH-SY5Y cells and rat brains. (**A**) The protein levels of Nrf2 and p-Nrf2 in the ipsilateral midbrain region were assessed with Western blotting. The blot is the representative of the two independent experiments. TATA box binding protein (TBP) was used as a loading control for nuclear fraction. (**B**) Cells were transfected with an ARE-luciferase construct (1 µg per well). After overnight, cells were treated with phloroglucinol or 6-OHDA, cell lysates were mixed with a luciferase substrate, and the luciferase activity was measured by a luminometer. (ANOVA, *post-hoc* by Duncan, * *p*<0.05, **p<0.01). (**C**) The protein levels of p-Akt and Akt in the ipsilateral midbrain region were assessed with Western blotting. The blot is the representative of the two independent experiments. The intensity of the bands was measured relative to the amount of Akt in each sample. The relative densitometric value of p-Akt *vs.* that of Akt is shown in graph.

The effects of treatment with 6-OHDA and the pre-treatment with phloroglucinol prior to 6-OHDA on Nrf2 and p-Nrf2 in the nuclear fraction were examined in SH-SY5Y cells. The treatment with 6-OHDA down-regulated the protein level of Nrf2 and p-Nrf2 in the nuclear fraction, while the pre-treatment with phloroglucinol reversed the reduction in Nrf2 and p-Nrf2 level in the nuclear fraction (Figure 7A).

Most of the genes encoding phase II detoxifying and antioxidant enzymes have an ARE sequence in their promoter region. Nrf2 is an important transcription factor that regulates ARE-driven antioxidant enzyme gene expression. To verify the functional relevance of Nrf2 binding to the ARE sequence of antioxidant enzyme, a luciferase activity assay was performed by using an ARE-luciferase gene containing the Nrf2 binding DNA consensus site linked to a luciferase reporter gene. As illustrated in Figure 7B, phloroglucinol restored the transcriptional activity of Nrf2 decreased by 6-OHDA significantly.

We also investigated the changes in the level of p-Akt in control, phloroglucinol, 6-OHDA and 6-OHDA plus phloroglucinol treated SH-SY5Y cells.

Phosphoatidylinositol 3-kinase (PI3K)/protein kinase B (PKB, Akt) are important signaling enzymes involved in transduction of various signals from the cell surface to the nucleus. This pathway is associated with the modulation of ARE-driven gene expression via Nrf2 activation [21]. We found that the treatment with 6-OHDA down-regulated the ratio of p-Akt vs. Akt, while the pre-treatment with phloroglucinol reversed the reduction in the ratio (Figure 7C).

## Discussion

In this study, we demonstrated that phloroglucinol, a component from the edible brown algae, *Ecklonia cava* can attenuate motor functional deficits as assessed with the rota-rod and apomorphine-induced rotation tests in a 6-OHDA-lesioned rat PD model (Figure 1). At 2 weeks post-injection of 6-OHDA, with or without administration of phloroglucinol into the right medial forebrain bundle, phloroglucinol was found to significantly prevent the reduction in the latency times to fall off the accelerating rota-rod (30.1±3.0 sec), compared with the 6-OHDA-treated group (13.2±1.6 sec, Figure 1A). In PD animal models prepared by administering toxins such as MPTP (1-methyl-4-phenyl-1,2,3,6-tetrahydropyridine) and 6-OHDA, motor functional deficits were assessed with a rotation test after subcutaneous administration of apomorphine or amphetamine. As shown in Figure 1B, we found that co-administration of phloroglucinol with 6-OHDA significantly attenuated the contralateral directional rotation against the 6-OHDA lesioned direction after subcutaneous injection of apomorphine (Figure 1B). Furthermore, we showed that phloroglucinol rescued the dopaminergic neuronal death and loss of synapses between dopaminergic neurons in the midbrain (Figure 2). The unilateral infusion of 6-OHDA into the medial forebrain bundle produced a significant reduction in the number of TH positive dopaminergic neurons/DAPI stained cells in the midbrain compared with the vehicle-treated control group (***p<0.001, Figures 2A, B), whereas the phloroglucinol treatment significantly prevented the loss of dopaminergic neurons as assessed with TH immunoreactivity and protein levels from 6-OHDA neurotoxicity (***p<0.001, Figures 2A, B).

Neural circuits of the basal ganglia are critical for motor planning and action selection [22]–[25]. Classically, it has been well established that Parkinsonian motor deficits result from an overactive indirect pathway and an underactive direct pathway caused by dopaminergic neuronal death that occurs in the *Substantia nigra* [22]. Dopamine is released into the striatum from neurons located in the *Substantia nigra* and exerts a critical role for the modulation of basal ganglia activity, where alterations in this system are implicated in the pathophysiology of PD [26].

Although the exact cause of the selective cell death in PD is largely unknown, multiple lines of evidence suggest an important role for oxidative stress. Dopaminergic neurons are particularly prone to oxidative stress due to dopamine metabolism and auto-oxidation combined with increased iron, decreased total glutathione levels and mitochondrial complex I inhibition-induced ROS production in the *Substantia nigra* which can lead to cell death by exceeding the oxidative capacity of dopamine-containing cells in the region [13]. Based on studies of the actions of toxins, postmortem investigations, and genetic defects involved in familial PD, there is now a general consensus about the mechanisms of cell death that contribute to the neuronal loss observed in PD [22]. Among the several mechanisms such as mitochondrial dysfunction, oxidative stress, altered protein processing and inflammatory changes, considered to be associated with PD pathogenesis [22], mitochondrial dysfunction and oxidative stress are thought to be critical factors for the disease [27]–[32].

Previously, we reported that phloroglucinol, a phlorotannin compound isolated from *Ecklonia cava*, protects against H_2_O_2_-induced cell damage through the activation of an antioxidant system in *in vitro* [7], and gamma-ray radiation-induced oxidative damage *in vitro* and *in vivo* [35].

In this study, we demonstrate that phloroglucinol reduces intracellular ROS, lipid oxidation, protein carbonylation and DNA oxidation induced by the treatment with 6-OHDA in SH-SY5Y cells. Furthermore, phloroglucinol restored the decrease in the enzyme activities and the protein levels of antioxidant enzymes, (i.e. catalase and glutathione peroxidase) in *in vitro* and *in vivo* PD experimental system. The rescue of the protein level of catalase and glutathione peroxidase reduction by 6-OHDA was substantiated in rat PD models (Figures 6E, F).

Nrf2 is a basic-leucine zipper transcription factor that is ubiquitously expressed at low levels in all human organs. As Nrf2 regulates a major cellular defense mechanism, tight regulation is crucial to maintain cellular homeostasis. In cells, Nrf2 is constantly controlled by the repressor protein Kelch-like ECH-associated protein-1 (Keap1), which is a special molecular ‘sensor’ of changes in intracellular homeostasis. A constant feature of these molecular structures unites them into the single redox-sensitive signaling system Keap1/Nrf2/ARE whose main purpose is to maintain intracellular homeostasis under exposure to apoptosis-inducing and oncogenic factors and stress. ARE controls the expression of more than 100 genes, including heme oxygenase 1 and glutathione peroxidase. Under normal physiological conditions, Nrf2 binds to Keap1 and is thereby sequestered in the cytoplasm in association with the actin cytoskeleton [36], [37]. Under electrophilic, mild oxidative-stressed conditions, free Nrf2 released from Keap1 is up-regulated as a cellular adaptive response, whereas in severe oxidative-stressed conditions induced by strong oxidant molecules such as 6-OHDA, the level of Nrf released from Keap1 is decreased. Consequently, cellular antioxidant enzyme activity is down-regulated [19], [20], [38], [39]. Nuclear translocation of Nrf2 has been reported to be modulated by phosphorylation via several kinases including ERK1/2 and PI3K/PKB [40], [41].

Here, we found that the pre-treatment with phloroglucinol rescued the reduction in Nrf2 and p-Nrf2 in the nuclear fraction induced by 6-OHDA treatment (Figure 7A). Modulation of Nrf2 has been shown in several neurodegenerative disorders. The overexpression of Nrf2 has become a potential therapeutic avenue for various neurodegenerative disorders such as Parkinson, amyotrophic lateral sclerosis, and Alzheimer's disease.

Based on our results, we infer that the antioxidant properties of phloroglucinol may result from three action mechanisms as follows. First, phloroglucinol has a polyphenolic structure and is an electron-rich compound that is prone to enter into efficient electron-donation reactions with oxidizing agents to produce intermediate phenoxyl radical (PhO·) species. Phenoxyl radicals are stabilized by resonance delocalization of the unpaired electron to the *ortho* and *para* positions of the ring. In addition to the resonance stability, phenoxyl radicals can be stabilized by hydrogen bonding with an adjacent hydroxyl group. Phenoxyl radicals also undergo dimerization (“phenol coupling”) to produce new CC or CO linkages [7]. The intrinsic stability of phenolic structures might be related to the antioxidative activity of phloroglucinol. Second, phloroglucinol can up-regulate the expression of antioxidant enzymes such as catalase and glutathione, by increasing the transcriptional activity of Nrf2. Third, phloroglucinol can increase the enzyme activities of antioxidant enzymes (catalase and glutathione peroxidase), although further study is needed for elucidating the mechanisms.

Increasing populations worldwide suffer from PD due to an extended life span. However, fundamental therapeutics or treatment strategies have not been available until now. Much effort has been given towards the development of therapeutics, with the hope to prevent the selective degeneration of dopaminergic neurons in the *Substantia nigra*. In the mid-1980s, the treatment of PD was exclusively centered on L-dopa therapy and focused on dopamine systems and motor symptoms. In the early 2010s, novel dopamine agonists (pramipexole, ropinirole, rotigotine), catechol methyltransferase inhibitors (entacapone), and monoamine oxidase B inhibitors (rasagiline) were developed to provide more continuous oral delivery of dopaminergic stimulation to improve motor outcomes. Despite these therapeutic advances, PD continues to be a relentlessly progressive disorder that leads to severe disability. Neuroprotective interventions are able to modify the progression of PD, but have stood out as a failed therapeutic goal over the last 2 decades, despite potentially encouraging results from compounds like rasagiline [13].

In this study, we administered phloroglucinol directly into the brain by using a stereotaxic apparatus. Pharmacokinetic studies remain to be performed to examine whether phloroglucinolcan pass through the BBB.

Taken together, the present study demonstrates the therapeutic potential of phloroglucinol for the treatment of PD.
